# Supplementary material for: AMDDLmodel: Android smartphones malware detection using deep learning model
Source: PLoS One. 2024 Jan 19;19(1):e0296722. doi: 10.1371/journal.pone.0296722 (PMC10798489; doi:10.1371/journal.pone.0296722)
Supplement: Attachment — Submitted filename: Responses_to_Reviewer-2.docx [file pone.0296722.s001.docx]

**Reviewer 2 Comments and Suggestions**

**Manuscript Number:** PONE-D-23-25528

**Title:** **AMDDLmodel: Android Smartphones Malware Detection Using Deep Learning Model**

Dear Editor-in-Chief and Distinguished Reviewers,

We are extremely grateful to all the reviewers for providing us with constructive feedback and we highly appreciate the suggestions and criticisms made in order to strengthen the manuscript. We have taken all comments into consideration and provided our itemized response. With these changes, we sincerely wish that our manuscript now be considered for acceptance in “PLOS ONE”. Please note that responses to your comments are given in the refined version of the manuscript.

**Responses to Reviewer’s Comments:**

**1. The paper shows no novelty**

Respected Reviewer,

Please find the changes in red color in the refined version of the manuscript.

**[Thank you for your valuable comments. The comment has been addressed into introduction section and highlighted in red color as “The novelty of our research lies in the development of the AMDDLmodel, a unique deep learning model tailored specifically for Android malware detection. Unlike previous works, our model offers improved accuracy and efficiency in identifying malware on Android smartphones. By leveraging deep learning techniques and innovative feature engineering, we achieve superior performance in malware detection, which is a significant advancement in the field of mobile security.]**

**2. What are the contributions? Either data preprocessing, Novel model architecture??**

Respected Reviewer,

Please find the changes in red color in the refined version of the manuscript.

**[The key contributions of this research work are:**

1. **It introduces the AMDDLmodel, a novel deep learning approach tailored for precise Android malware detection.**
2. **The model enhances the accuracy and efficiency of malware identification on Android smartphones, thereby elevating device security.**
3. **The research incorporates inventive feature engineering techniques to extract meaningful insights from Android application data, a crucial component in accurate malware detection.**
4. **By addressing the real-world issue of Android malware, it provides users with practical tools to safeguard their devices.**
5. **A thorough evaluation of the model's capabilities and limitations, offering practical insights into its effectiveness for real-world implementation.]**

**3. No novel in methodology a simple CNN architecture.**

Respected Reviewer,

Please find the changes in the refined version of the manuscript.

**[Thank you for your valuable comments. The primary novelty in our research lies in the effective application and adaptation of this CNN architecture for Android malware detection. We've demonstrated that even with a straightforward architecture, the AMDDLmodel delivers highly competitive accuracy, outperforming state-of-the-art techniques. The significance of our approach lies in its practicality, efficiency, and its potential to benefit Android users by enhancing device security. While more complex architectures exist, the practicality and robustness of the AMDDLmodel make it a valuable contribution to the field of Android malware detection.]**

**4. Accuracy, loss not shown in tabular format.**

Respected Reviewer,

Please find the changes in red color in the refined version of the manuscript.

[**Thank you for your valuable comments. The accuracy and loss values have been updated in Table 1 and Table 2 and also highlighted in green color in the refined version of the manuscript.**]

**5. Comparison should be done based on all the metrics.**

Respected Reviewer,

Please find the changes in the refined version of the manuscript.

**[Thank you for your valuable comments. As the main concern and contribution in our model is to enhance the malware detection accuracy to secure the android devices, but the other statistical metrics like precision, recall and F1-score are also important and have some impact on evaluation scale. That’s why we consider only accuracy metrics to compare and conclude the results.]**

**6. No discussion section.**

Respected Reviewer,

Please find the changes in green color in the refined version of the manuscript.

**[Thank you for your valuable comments. The discussion section has been added to the refined version of the manuscript and also highlighted in green color.]**
